# Established binary cutoffs for cine-CMR basal slice selection - an unrecognized source of CMR discordance with echocardiography and necropsy derived LV mass

**DOI:** 10.1186/1532-429X-15-S1-P115

**Published:** 2013-01-30

**Authors:** Lauren A Simprini, Parag Goyal, Jamie Mullally, Noel Codella, Anika Afroz, Mitchell Cooper, David S Fieno, J Paul Finn, Richard B Devereux, Jonathan W Weinsaft

**Affiliations:** 1 Medicine/Cardiology, Weill Cornell Medical College, New York, NY, USA; 2 Medicine/Cardiology, Memorial Sloan Kettering Cancer Center, New York, NY, USA; 3 IBM Thomas J. Watson Research Center, Yorktown, NY, USA; 4 Heart South Cardiovascular Group, Alabaster, AL, USA; 5 Radiology, UCLA Health System, Los Angeles, CA, USA

## Background

Left ventricular mass (LVM) is widely used to guide clinical decision-making. CMR is well suited to measure LVM as it provides high-resolution delineation of myocardial contours. CMR quantification of LVM is typically performed via planimetry of contiguous short axis images, an approach fundamentally dependent on reader selection of short axis images to be contoured. Established methods have applied different binary cutoffs using circumferential extent of LV myocardium to define the basal LV, while omitting short axis images containing lesser fractions of LV myocardium. This study compared LVM, quantified using different established methods for basal slice selection, to independent references of LVM measured by echocardiography and necropsy.

## Methods

Cine-CMR (1.5T) was performed in patients and laboratory animals. Contiguous short axis SSFP images were acquired throughout the LV; myocardial circumference was quantified in all short axis slices. LVM was quantified with inclusion of all LV myocardium (ALL), and by two previously established methods that use different binary cutoffs to define the LV basal-most short axis slice: (1) 50% circumferential myocardium at end-diastole alone (ED50), (2) 50% circumferential myocardium throughout both end-diastole and end-systole (EDS50). Patient results were compared to LVM quantified by echocardiography performed within 1 day of CMR. Lab animal results were compared to LV weight at necropsy.

## Results

**Figure 1:**
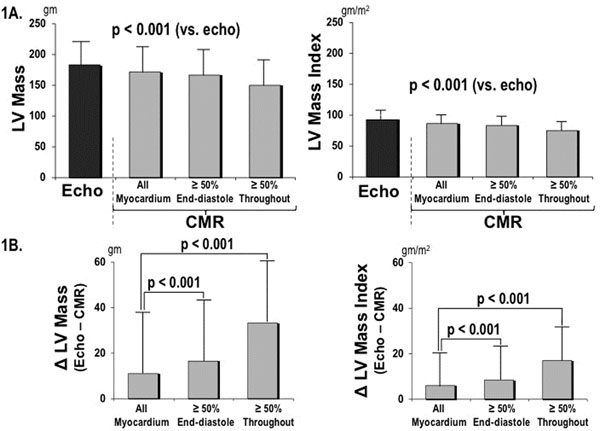
A) LV mass (mean ± SD) by each CMR basal slice selection method (gray bars) compared to echocardiography (black bar). B) LV mass difference between each CMR method and echocardiography, demonstrating smaller differences with inclusion of all myocardium as compared to each binary cutoff method (p<0.001).

**Figure 2:**
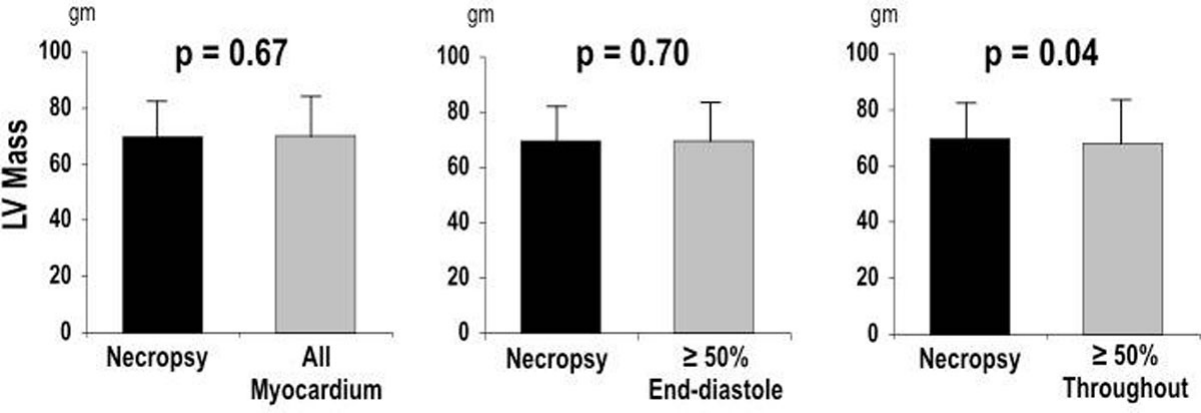
LV mass (mean±SD) by each CMR basal slice selection method (gray bars) compared to *ex vivo* LV weight at necropsy (black bar).

150 patients with CAD and 10 lab animals (8 dogs, 2 pigs) were studied. Among patients, methods discordantly assigned the basal-most short axis slice in nearly all exams (96%). In cases of methodological discordance, ED50 differed from ALL by ≥1 LV short axis slice in 48%, and EDS50 differed from ALL by ≥2 slices in 52% of exams. Compared to LVM by ALL (172.6±42.3 gm), LVM was significantly lower (p<0.001) when quantified by either ED50 (167.2±41.8 gm) or EDS50 (150.6±41.1 gm). In patients (Figure 1), ALL yielded smaller differences with echocardiography (Δ=11.0±28.8 gm) than did ED50 (Δ=16.4±29.1 gm) and EDS50 (Δ=33.2±28.7 gm, both p<0.001). In lab animals (Figure 2), *ex-vivo* LV weight (69.8±13.2 gm) was similar to LVM calculated using ALL (70.1±13.5 gm, p=0.67) and ED50 (69.4±13.9 gm, p=0.70), whereas EDS50 (67.9±14.9 gm, p=0.04) yielded small but significant differences with LV weight at necropsy.

## Conclusions

Established CMR methods yield frequent discordance regarding assignment of the basal-most short axis LV slice, resulting in significant differences in calculated LVM. Inclusion of all myocardium, rather than use of binary cutoffs for basal slice selection, yields smallest CMR discrepancy with echocardiography measured LVM and non-significant differences with necropsy measured LV weight.

## Funding

K23 HL102249-01, Lantheus Medical Imaging (unrestricted research grant)

